# Cardiac magnetic resonance characteristics of pediatric Cardiac Teratoma

**DOI:** 10.1186/1532-429X-14-S1-T2

**Published:** 2012-02-01

**Authors:** Amy L Tipton, Kan N Hor, Wojciech Mazur, David Collins, Melissa King-Strunk, William Gottliebson, Michael Taylor

**Affiliations:** 1 Heart Institute, Cincinnati Children's Hospital Medical Center, Cincinnati, OH, USA; 2 The Heart and Vascular Center, The Christ Hospital, Covington, OH, USA

## Summary

5 day old term female with prenatal history of an intracardiac tumor followed by a post-natal echocardiography indentifying a large cystic mass compressing both ventricles, but without significant outflow tract obstruction. Cardiac MRI imaging showed a very large ventricular septal cystic mass most consistent with a primary cardiac teratoma causing mild LVOT obstruction.

## Background

CMR is an established method for evaluation of cardiac masses. It is superior to other modalities as it can provide tissue characteristics, thus allowing highly accurate differentiation of benign from malignant tumors, as well as local mass effects such as outflow compression.

### Purpose

Using Cardiac MRI for assessment of etiology of a massive, septal tumor as well as its effect on LVOT flow and LV filling.

## Methods

A 5 day old full term female with a prenatal diagnosis of a large intracardiac tumor underwent post-natal echocardiography. Echo confirmed the suspected diagnosis as a large cystic mass compressing both ventricles without significant outflow tract obstruction. Cardiac MRI was performed specifically for further morphologic characterization. Imaging was performed using non-breatheld technique with multiple signal averages while under general endotracheal anesthesia on a 1.5 Tesla GE Excite magnet utilizing an 8-channel phased-array cardiac coil. The CMR sequences included SSFP 2, 3, and 4 chamber views and short axis multislice cine stacks; tagged SPAMM cines in 3 short and 2 long axis planes; gadolinium-enhanced myocardial first pass perfusion and delayed enhancement imaging in both short and long axis planes. Pre- and post-contrast T1 FSE single phase short axis stacks. Both right and left ventricular volumes and tumor volume were measured at end systole and end-diastole using MEDIS QMass software.

## Results

**Figure 1:**
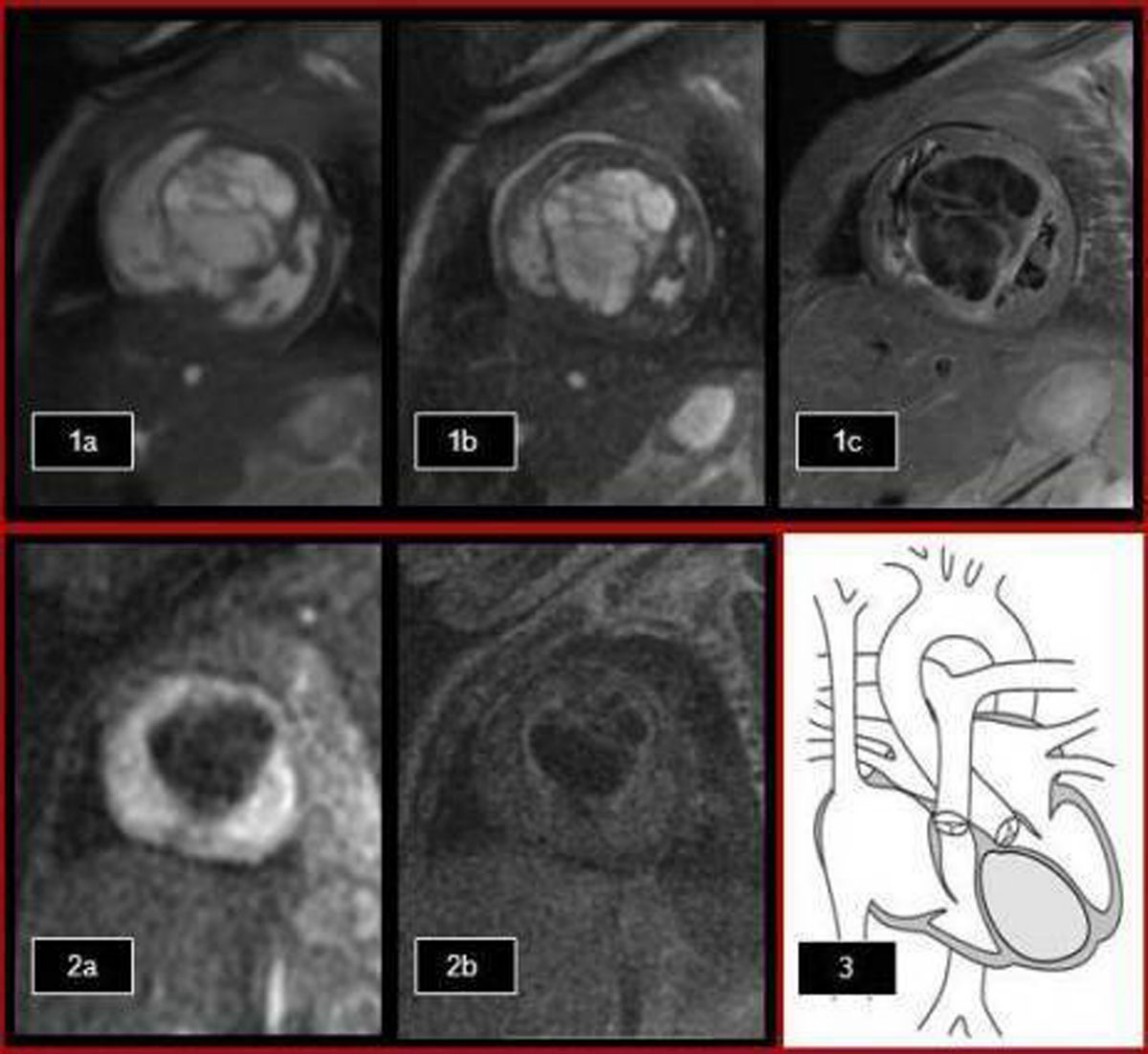


**Figure 2:**
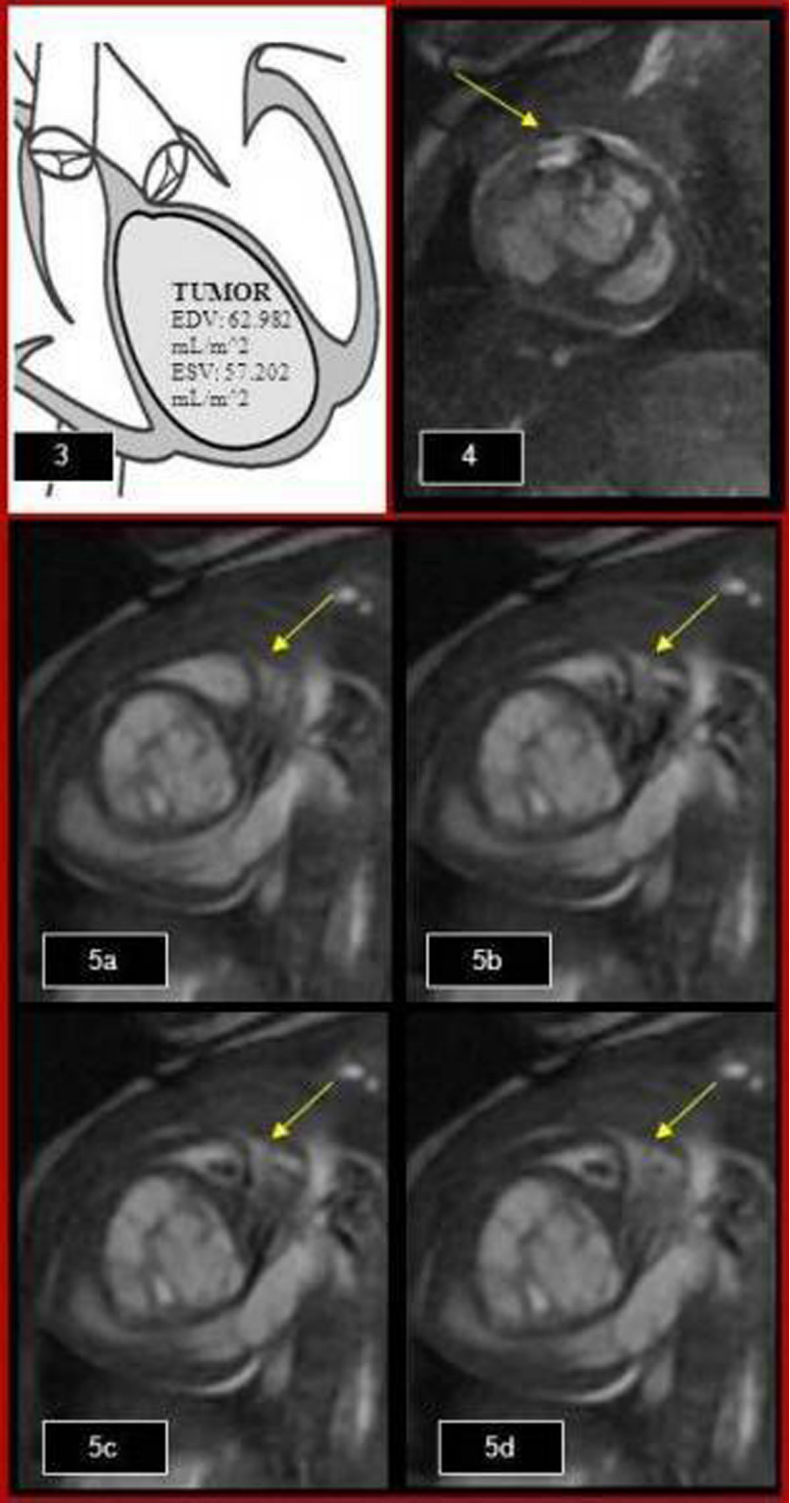


All the sequences show a large heterogeneous intraventricular cystic mass surrounded by thinned myocardium (Fig.1(a-c)). The tagged images shows the mass does not move with the contracting myocardial tissue. First pass perfusion (Fig. 1(2a)) and late gadolinium enhancement (Fig. 1(2b)) demonstrated no perfusion and no enhancement, confirming a vascular nature of the mass. Both ventricles despite compression, maintained normal systolic function (Fig. 1(3)). On the right side of the heart, the lesion compresses the RVOT (Fig. 2(4)) with trivial insufficiency of the tricuspid valve. There was turbulent flow in the left ventricular outflow tract consistent with mild obstruction (Fig. 2(5a-d)). Given the tissue characteristics and typical location, diagnosis of cardiac teratoma was confirmed.

## Conclusions

CMR provided exquisite anatomic localization and tissue characterization of this pediatric cardiac tumor. It also delineated the extent of viable function within the heart chambers allowing for proper patient management. Based on the MR imaging, no interaction was undertaken. At the time of the last evaluation at 4 months of age, the patient was doing well and was without symptoms. The tumor size was unchanged and the cardiac function remains normal.

## Funding

There are no financial disclosures.

